# Genetic characterization and mutation analysis of Qihe547 Aujeszky’s disease virus in China

**DOI:** 10.1186/s12917-018-1492-2

**Published:** 2018-07-06

**Authors:** Cun Liu, Yanhan Liu, Ye Tian, Xuehua Wei, Yue Zhang, Fulin Tian

**Affiliations:** 1 Shandong Provincial Center for Animal Disease Control and Prevention, Ji’nan, 250022 Shandong China; 2 College of Veterinary Medicine, China Agricultural University, Beijing, 100193 China

**Keywords:** Aujeszky’s disease virus, Qihe547, Phylogenetic analysis, Virus isolation

## Abstract

**Background:**

Aujeszky’s disease virus (ADV) can cause neurologic disease in young pigs, respiratory disease in older pigs and abortion or birth of mummified fetuses or stillborn neonates. The re-emergence of Aujeszky’s disease (AD) in pig farms vaccinated with live vaccine (Bartha-K61) caused substantial economic losses to Chinese pig industry since late 2011. A field ADV, named Qihe547, was isolated from pigs that exhibited suspected AD clinical symptoms. To better understand the genetic characteristics and mutations of Qihe547 ADV, the whole genome was sequenced and analyzed.

**Results:**

The genomic length of Qihe547 ADV was 143,404 bp, with 73.59% G + C contents. Phylogenetic analysis based on the whole genome of ADV strains revealed that Chinese ADV strains were located to one group with three subgroups. Qihe547 ADV was closely related to these novel ADV strains isolated in China since 2012. Qihe547 presented numerous hypervariable regions compared with oversea ADV strains. In 34 genes of Qihe547 ADV, amino acid (AA) insertion or deletion were observed. In addition, numerous AA mutations were found in the main protective antigen genes (gB, gC and gD genes). The differences of potential antigenic peptides in the main protective antigens between Qihe547 ADV and ADV Bartha were discovered in the dominant antigenic regions of gB (AA59-AA126, AA507-AA734),the extracellular region of gC and gD.

**Conclusion:**

High diversity was observed between Qihe547 and foreign ADV isolates. The AA variations and the differences of potential antigenic peptides in the important functional regions of the main protective antigen (gB, gC and gD) of ADV Qihe547 may contribute to immune evasion of the virus and may be partial reason that the virus escapes from the vaccination of Bartha-K61 vaccine. In a word, the effect of the variations obviously requires further research.

## Background

Aujeszky’s disease (AD), a devastating swine disease, also known as Pseudorabies (PR), is an economically important viral disease of swine worldwide [1–3]. AD is characterized by high morbidity in piglets, severe respiratory disorders in older pigs and sow’s reproductive failure [2, 4]. AD occurs all over the world and caused great economic losses to the pig industry in many countries [5].

The causative agent responsible for AD is Aujeszky’s disease virus (ADV) which is a member of the genus *Varicellovirus* of the subfamily *Alphaherpesvirinae* within the family *Herpesviridae* [6]. The genome of ADV is double-stranded DNA, approximately 150 kb, with G + C contents more than 70%. Swine are the natural host for ADV and the reservoir of the virus in nature [7], but a wide range of mammals can be infected with ADV [8].

Vaccination is widely used to control the AD. Attenuated live vaccine has been widely used for many years in many countries to diminish the economic losses caused by AD [9]. Due to large-scale compulsory vaccination with gE-deleted vaccines and the DIVA (differentiating infected from vaccinated animals) strategy, AD has been eradicated from domesticated pigs in many countries [3, 10]. In the 1970s, China imported the Bartha-K61 vaccine from Hungary [11]. Then, it was widely used in swine farmers, from the 1990s until late 2011, AD well controlled. Since late 2011, however, AD broke out in China and has caused huge economic losses [11, 12]. It is great necessary to understand the genetical mutation of re-emergenced ADV strain.

In this study, we genetically characterized Qihe547 ADV strains from swine herd vaccinated with live vaccine by the Sanger dideoxy-chain termination method and phylogenetic analysis to get a better insight into the genetic relatedness between strains in both populations and to compare Qihe547 strains with strains circulating in neighboring countries. To understand the mutations of Qihe547 ADV and the causes of immune failure, comparative genomic analyses and phylogenetic analysis were performed.

## Methods

### Ethics and consents

Sampling was performed under the permission from the owner of the swine herd in Qihe, Shandong province, China and the ethical approval was granted by the Administration Bureau of Animal Husbandry and Veterinary Medicine of Shandong. Animal manipulation procedures adhered to the guidelines under the Animal Ethics Committee of the Administration Bureau of Animal Husbandry and Veterinary Medicine of Shandong.

### Samples collection

In 2014, outbreak of AD occurred in a pig farm in Qihe, Shangdong province, China. In the farm, the pregnant sows showed high rates of abortion and some fattening pigs showed severe respiratory symptoms along with high fever and then died. The tissue samples, including brain, lung, kidney, liver, and lymph node, were collected from the clinical dead pigs for diagnostic tests and the corpses were harmlessly treated after anatomical examination. The clinically dead pigs were from the swine herd in Qihe area, Shandong province, China. The tissue samples were homogenated. The homologized samples were centrifuged at 8000 g for 5 min. Then, the supernatant was used to extracted DNA and virus isolation.

### Virus isolation and identification

Nucleic acid was extracted from the supernatant using AxyPrep Body Fluid viral DNA/RNA Mini-prep kit according to the manufacturer’s protocol (Corning Co., China). The nucleic acid was detected for Aujeszky’s disease virus (ADV), porcine reproductive and respiratory syndrome virus (PRRSV), porcine circovirus type 2 (PCV2), swine fever virus (SFV) by the real-time PCR. The supernatant, ADV positive, was filtrated through 0.22 μm filter (Millipore, Milford, MA) and inoculated to monolayer culture of Monolayer culture of porcine kidney 15 (PK-15) cells cultured in dulbecco’s modified eagle mediun (DMEM) (Hyclone) supplemented with 8% fetal bovine serum (Shanghai ExCell Biology Co.). PK-15 cells were maintained at 37 °C in an atmosphere of 5% CO2. The cytopathic effect (CPE) was examined daily.

Then, the virus was identified by indirect immuofluorescence assay (IFA). Briefly, inoculated cells were fixed with cold absolute ethanol for 1 h, blocked with 1% bovine serum albumin (Sigma, St. Louis, MO) for 30 min, and incubated with 200 times diluted Rabbit anti-ADV antibody (Abcam Ab3534) for 1 h, followed by a 2000 dilution of goat anti-rabbit IgG-FITC-conjugated secondary antibody (Abcam Ab6717) for 1 h. Cell staining was examined under a fluorescence microscope. Finally, the ADV was plaque-purified three times and propagated on PK-15 cells. Virus titer was determined by measuring the 50% tissue culture infective dose (TCID50).

### PCR and sequencing

Primers for the amplification of Qihe547 ADV whole genome were designed based on ADV Becker (JF797219). The plaque-purified virus was used to extract DNA with the AxyPrep Body Fluid viral DNA/RNA Mini-prep kit according to the manufacturer’s protocol (Corning Co., China). PCR was performed with the Takara LA Taq with GC buffer kit (Takara, Dalian), following the manufacturers’ instructions. Cycling conditions for PCR was as follows: denaturation at 95 °C for 10 min, followed by 38 cycles of 95 °C for 1 min, gradiently annealing temperature 50 °C~ 70 °C for 50 s, and 72 °C for 1 min, and final extension of 72 °C for 10 min. The products were cloned into pMD18-T vector (Takara, Dalian). The recombinant plasmid, containing the target DNA fragments, was sent to BioSune Biotech Co. Ltd. (Jinan, China) for DNA sequencing by the Sanger dideoxy-chain termination method after restriction endonuclease digestion with EcoRI and Hind III.

### Sequence assembling and alignments

Sequences of the target DNA fragments were assembled using SeqMan (DNASTAR, Madison, WI). Gene’s annotation of Qihe547 ADV was based on ADV Becker and ADV JS-2012(KP257591). The alignment based on full-length genome sequences between Qihe547 ADV and reference ADV strains from GenBank was performed using the mVISTA genomics analysis tool with global LAGAN alignment [13].

Each gene’s protein-coding sequence was filtered out from the whole genome sequence of Qihe547 ADV and foreign ADV strains (ADV32751/Italy2014, Kolchis, Bartha, Becker, and Kaplan). To study the deduced amino acid (AA) sequence mutations in each genes of Qihe547 ADV relative to foreign ADV isolates (ADV32751/Italy2014, Kolchis, Bartha, Becker, Kaplan), AA sequence alignments were performed. Alignments of the deduced amino acid sequence of each gene’s protein-coding sequence were performed with MegAlign (DNASTAR, Madison, WI). The potential antigenic peptides in glycoprotein (g) B, gC, gD of Qihe547 ADV and Bartha were predicted by Antigenic Peptide Prediction online tool (http://imed.med.ucm.es/Tools/antigenic.html).

### Phylogenetic analyses

The phylogenetic analysis was performed by the neighbor-joining method. The phylogenetic tree based on full-length ADV genome sequences and gB, gC and gD genes sequences were constructed by MEGA 6.0 software with 1000 bootstrap replication.

## Results

### Virus isolation and identification

**Fig. 1:**
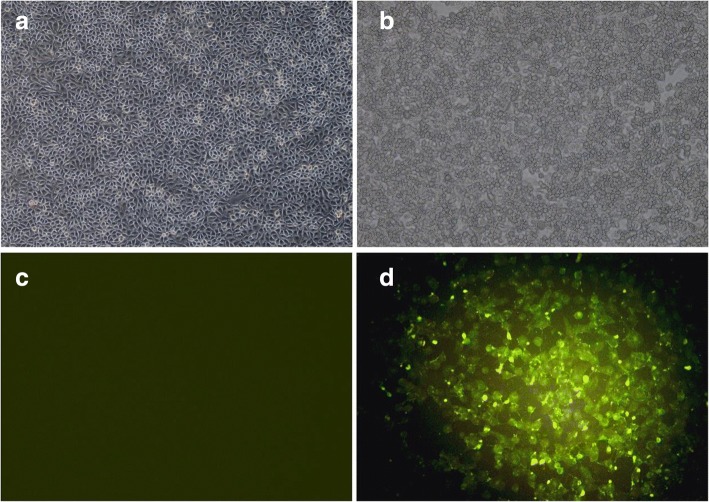
The indentification of Qihe547 ADV. **a** and **c.** Negative control, PK-15 cells uninfected with Qihe547 ADV. **b.** Specific cytopathic effects of Qihe547 ADV (× 100). **d.** Indirect immunofluorescence assays of Qihe547 ADV in PK-15 cells (× 200)

ADV-positive mixed tissue sample was confirmed by the real-time PCR and the supernatant inoculated onto PK-15 cells. A distinct CPE was observed (Fig. 1b) and identified with IFA (Fig. 1d). The isolated ADV was named Qihe547. The titers of Qihe547 ADV were 10^8.0^TCID_50_/ml.

### Genomic characterization of ADV Qihe547

**Fig. 2:**
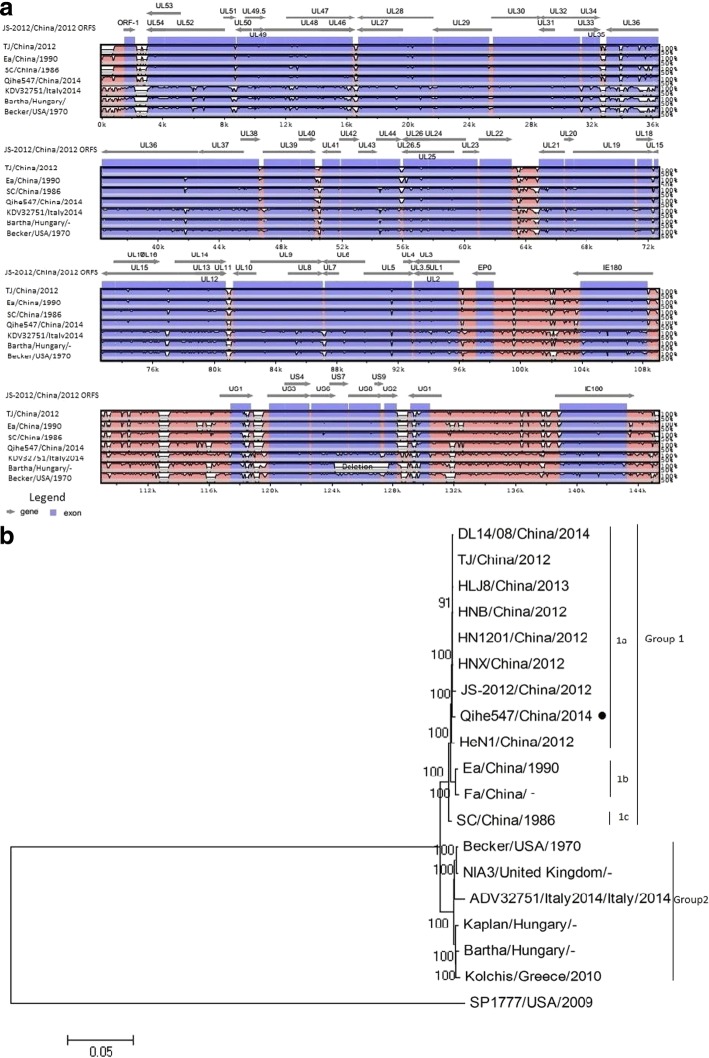
Comparison and phylogenetic analysis based on the whole-genome sequences of ADV strains. **a.** The multiple sequence alignments showed the conserved regions within Qihe547, TJ, Ea, SC, ADV32751/Italy2014, Becker, Bartha strains. The conservation score was plotted in a sliding 100 bp window. **b.** Phylogenetic tree based on whole-genome sequences of PRV strains was constructed using MEGA 6.0 software. SP1777, a bovine herpesvirus strain, served as an outgroup. Bootstrap values obtained from 1000 replicates are shown at node points. Qihe547 isolated in this study was indicated by a solid dot

The genome of Qihe547 ADV was sequenced and approximately 143 kb long. Its G + C contents was 73.59%. Its genome organization was similar to other ADV strains submitted to GenBank. The genomic annotation was performed according to the sequences information of ADV Becker and JS-2012. Genome sequence of Qihe547 ADV submitted to Genbank, the accession No. is KU056477. Interestingly, compared to other ADV isolates from GenBank, there are special polynucleotide sequences (5’ CCCAGCTCTCCCCCGAGGGCCCAGCTCTCCCCC 3′) at the genome 5′- terminal. Upstream gB gene, the unique repeats region, possible repeating unit (5’ GGGGAGAGGGGAGACGAGA3’) different from ADV Becker (5’GGGACGGAGGGGAGA3’), exists a gap. Multi-genome alignments based on ADV isolates indicated that numerous hypervariable regions were found between Qihe547 ADV and oversea ADV isolates (ADV32751/Italy2014, Batha, Becker), especially in intergenic sequences (Fig. 2a). However, between Qihe547 and other China ADV isolates (JS–2012, TJ, Ea, SC), the genetic diversity showed little differences.

### Multiple sequences comparison

**Fig. 3:**
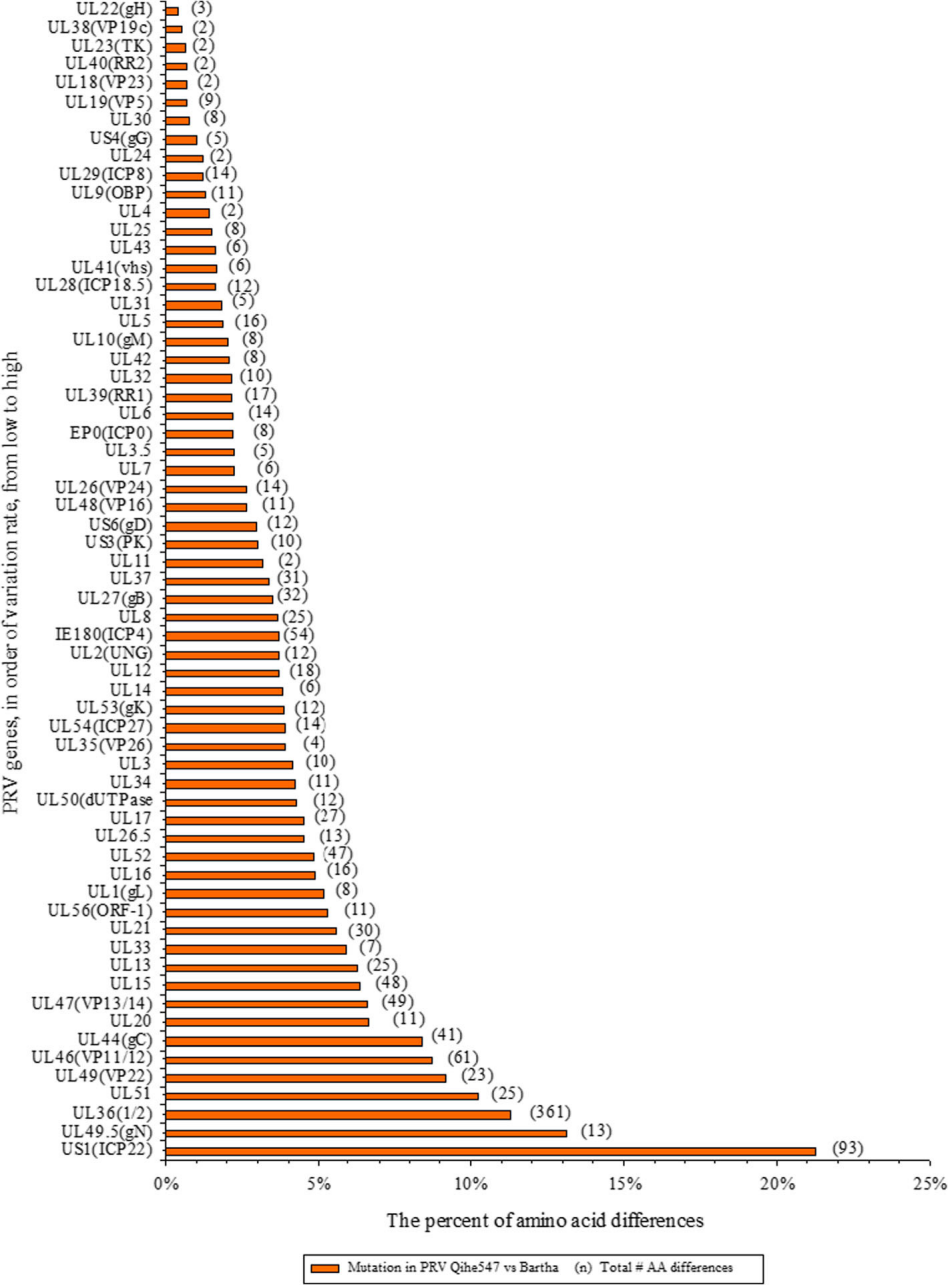
Protein-coding variation in Qihe547 ADV versus Bartha ADV. The bars showed the percent of amino acid differences in Qihe547 ADV versus Bartha ADV. Protein names were listed on left. Four proteins (US7, US8, US9, and US2 proteins) were absent because of the deletion regions in the unique short region of Bartha ADV

Compared with foreign ADV isolates (ADV32751/Italy2014, Kolchis, Bartha, Becker, Kaplan), a large amount of AA substitutions were observed in nearly each genes of Qihe547. Surprisingly, numerous unique AA indels were also found in Qihe547 genes (Table 1). The indels were observed mainly in genes associated with viral egress, viral replication and gene regulation. What’s interesting is that the unique indels in subunits of primase complex, products of UL52, UL5 and UL8 genes and these variations closely related to repeating sequences. To the contrary, Qihe547 ADV showed high level identities with other Chinese ADV isolates in protein sequences. But, analysis of variation between Qihe547 and other Chinese ADV strains showed that some genes had a single AA insertion or deletion, IE180 gene sequence a insertion (456S) and a deletion (98Q), UL46 gene sequence one deletion (571E),UL3 gene sequence a insertion (101A). Compared with ADV Bartha, high diversity was observed in Qihe547 ADV genes (Fig. 3). The highest divergence showed in the US1 gene. Moreover, the divergence in UL36, UL49.5 and UL51 genes were more than 10%. Compared with foreign ADV strains (ADV32751/Italy2014, Kolchis, Bartha, Becker, Kaplan), numerous AA variations were discovered in gB, gC and gD of ADV Qihe547, there being also AA consistent with ADV Becker or ADV ADV32751/Italy2014 (Table 2). Interestingly, there were AA differences between Chinese ancient ADV strains (Ea, Fa, SC) and novel ADV ADV strains (Qihe547, DL14/08, HeN1) isolated since 2012, such as R454K, H563Q, V740A in gB, G194E in gC and V338A in gD. Compared with Chinese ancient ADV isolates (Ea, Fa, SC), there were two AA deletion (^278^(R/S) P^279^) in gD of three novel ADV isolates. In gD of ADV LA and MinA, another two Chinese ancient ADV strains, there are four AA deletion at the same position. Between Qihe547 ADV and Bartha, the potential antigenic sites differences in the main protective antigen genes, gB, gC and gD genes, were found (Table 3). In AA59-AA126 of ADV Qihe547 gB gene, there were one more potential antigenic sites and three potential antigenic sites compared to ADV Bartha. In the N-terminal AA43-AA59 of Qihe547 ADV gC gene, two potential antigenic sites were predicted with the opposite to ADV Bartha a single potential antigenic site at the same position. Similarity, in the N-terminal AA62-AA88 of Qihe547 gD gene, there were two potential antigenic sites, one predicted at the same position of Bartha gD gene.

**Table 1:** Amino acid Indels in Qihe547 ADV genes, compared to other ADV strains

Name	Indels	Name	Indels
UL52	428-432(+QAHSQ), 596-597(−AL), 622(+V), 663-665(+SSS)	UL51	196-200(+EADAE), 223-234(+KK)
UL50	21(+R)	UL49.5	5(+S)
UL47	71-75(−(E/R)(E/G)ER(M/T)), 128(−E), 224(+L)	UL46	502-503(-PP), 568-570(+EEE), 588-592(+GNAAD), 618-621(-GSFR)
UL44	63-69(+AAASTPA)	UL42	439(+S)
UL39	15-16(−(A/P)P)	UL33	9-10(+GG)
UL32	221(+G)	UL29	345(+G), 1123(+G), 1140(+G)
UL28	429-430(+GA)	UL27	92(+I)
UL26	372-375(+GLPP),457-458(+AA)	UL26.5	126-129(+GLPP), 211-212(+AA)
UL25	242(+P), 318(+D)	UL21	364-368(+NGDGG),410-414(+PIVSA)
UL20	7(+V), 19-21(+AAV)	UL17	254-255(+PR)
UL13	28-34(-GGAIAAA)	UL9	285(+T)
UL8	121(+V), 134(+K), 142-144(+DED)	UL6	8(+A), 451(+R)
UL5	2(−A), 579-591(+PGGPGGPGGAPAS)	UL3	95-98(+TTTT)
UL2	51-56(+GAGAGA)	EP0	187(+R)
IE180	27-28(+AA), 900-906(+SPGTKSG)	US7	178(-S), 238(+G)
US8	48(+D), 496(+D)		
UL15	168(+A), 189-190(-GP), 200-203(+RGES), 214-218(+GSGAK), 648-649(+PG)	US1	343-398(+EEDEEEEDEEEEDEEEEDEEEEDEEEEDEEEEDEEEEDEEEDEEEEDEEEEDEEE)
UL36	249-257(+GAPAVAAVG), 562-563(−GA), 2256-2269(+APPAEAAPAAKPAP), 2339-2347(+RAPPPPQPQ), 2368-2392(+PPPQQQQQQQQQRQEPPAATASKKA), 2449-2456(+TQPAAPAE), 2476-2478(+TAK), 2736(+S), 2911(+V), 2973-2974(+PA), 3040-3045(+RAEPAR), 3056(+E), 3110-3111(+PF), 3126-3128(+SPL), 3142(+E), 3211(+P)		

+: Amino acid insertion

-: Amino acid deletion

**Table 2:**
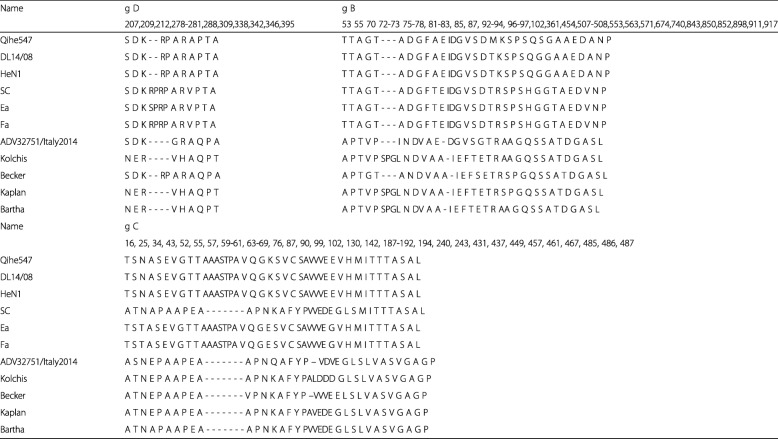
Amino acid mutation in gB, gC and gD of Qihe547 ADV

**Table 3:** The differences of the potential antigenic peptide in gB, gC and gD between Qihe547 and Bartha ADV. In the N terminus of ADV gB, there are antigenically important regions, such as (AA59-AA126, AA214-AA279, AA507-AA734) [21]. The extracellular region (AA23-AA453) of ADV gC plays an important role in ADV adsorption, neutralizing antibody generation and virus neutralization. gD also is the main target of ADV antibody. Mutation occurred in these regions may result antigen drift. Numerous AA mutations and potential antigenic peptides differences were found in gB, gC and gD of Qihe547, this may lead to immune failure

Name	Qihe547^a^	Bartha^b^
gB	^89^IDGAVSP^95^	^65^ASASPTPVPGSP^76^ ^79^TPNDVSAE^86^ ^433^EAIDAIYQ^440^
gC	^43^AGELSPSP^50^ ^52^STPEPVSG^59^	^43^AGELSPSPPPTPAPA^57^
gD	^62^LEDPCGVVALISDPQVDR^79^ ^81^LNEAVAHR^88^	^62^LEDPCGVAALISDPQVDRLLSEAVAHR^88^

^a^the potential antigenic peptide only in Qihe547

^b^the potential antigenic peptide only in Bartha

### Phylogenetic analyses

**Fig. 4:**
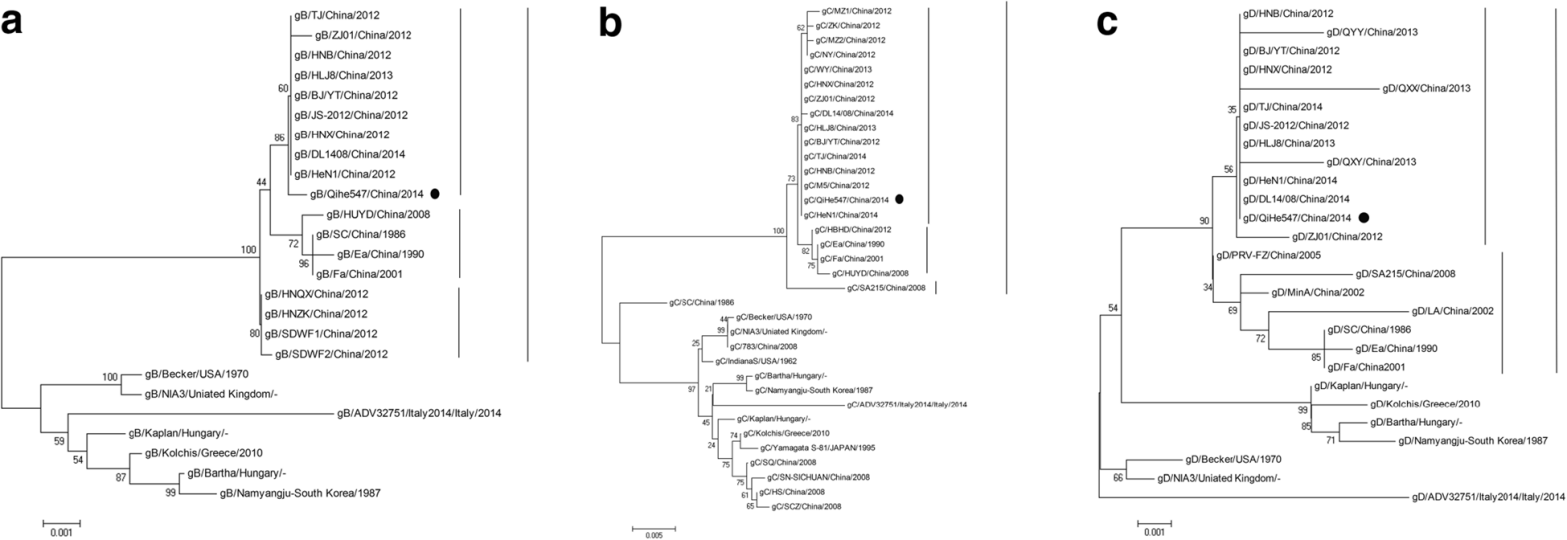
Phylogenetic analysis of gB, gC and gD genes. Phylogenentic trees were constructed by Neighbour-joining method using MEGA 6.0 software. Bootstrap values obtained from 1000 replicates were shown at node points. Qihe547 ADV was indicated by a solid dot. **a.** Phylogenetic tree of gB gene. **b.** Phylogenetic tree of gC gene. **c.** Phylogenetic tree of gD gene

Phylogenetic analysis based on completed genome sequence indicated that all ADV isolates used to construct phylogenetic tree were divided into two groups (group 1 and group 2) (Fig. 2b). All Chinese ADV isolates were located in group 1, and subdivided into group 1a, group 1b and group 1c. Group 1a comprised ADV strains isolated since 2012 (e.g., Qihe547, DL14/08, TJ, HeN1, HNB, HNX). Group 1b contained two Chinese traditional ADV isolates such as Ea and Fa. Another traditional ADV isolate SC belonged to group 1c. It indicated that Qihe547 ADV had close relationship with Chinese ADV strains isolated since 2012. Phylogenetic trees of gB, gC and gD indicated that nearly all Chinese ADV strains were located in one group (Fig. 4). In the phylogenetic trees of gC gene, six Chinese ADV isolates (SC, 783, SQ, SN-SICHUAN, HS, and SCZ) were clustered together with foreign isolates. This result showed that these strains were closely related to foreign ADV strains. Interestingly, phylogenentic relationship of SC ADV in the phylogennetic tree of gC was different from that in the phylogennetic tree based on completed genome sequences. In the phylogenentic of gD, Becker and NIA3 strains were clustered together with Chinese ADV isolates. Both genome-wide and gB, gC and gD genes phylogenetic analysis showed that Chinese ADV strains isolated since 2012 were not located in same subgroup with these strains isolated before 2012.

## Discussion

AD caused substantial economic losses to the pig industry in many countries, especially in regions with dense pig populations [10]. Although it has been eradicated from domesticated pigs in many countries, AD remains one of the most important diseases of swine. Mounting evidences showed that ADV infections are more widespread in wild animals and it is a threat to domestic pigs [2, 14–16]. In China, tremendous progress has been made in controlling and eliminating ADV in herds, including large-scale vaccination with live vaccine from the 1990s until late 2011. Since late 2011, the re-emergence of AD in pig farms has attracted the attention of researchers. In this study, Qihe547 strain, a field Aujeszky’s disease virus, was isolated and identified from herd vaccinated with live vaccine (Bartha-K61). For a better understanding of Qihe547 ADV genome characteristic, the full-length genome was sequenced.

Similar genome organization, with 73.59% G+C contents, were observed. The differences between Qihe547 and foreign ADV isolates distributed whole-genome-widely. Some interesting regions were found in Qihe547 ADV genome sequence, such as the unique upstream repeat region of gB gene which the possible repeat unit was different with Becker strain. Further study was needed to research the function of the repeat region for the virus. According to the phylogenetic analysis, Qihe547 ADV within subgroup 1a was closely related to these novel ADV strains isolated since 2012. Compared with foreign ADV strains (ADV32751/Italy2014, Kolchis, Bartha, Becker, Kaplan), unique AA indels were observed in 34 genes of Qihe547 ADV, mainly in proteins associated with viral egress, viral replication and genetic regulation. The highest divergence (more than 20%) was found in US1 protein acting as regulatory protein between Qihe547 ADV and Bartha. UL49.5 protein (gN) associated with immune evasion and UL36 and UL 51 proteins associated with viral egress also showed higher variation rates. Previous studies also draw similar conclusions [17]. Varying degrees of variation were also found in other proteins.

Mutations in antigen contribute to immune failure. Glycoprotein gB, gC and gD, the main protective antigen of ADV, could induce protective immune responses. The variability of gB, gC and gD was beneficial to ADV evading the host immune defense mechanism [18]. The differences displayed on glycoproteins of gB, gC and gD between ADV vaccine strain and prevalent ADV strains might contribute to immune failure. Vaccination or challenge experiments in sheep and pigs indicated that Bartha-K61 vaccine could not provide complete protection against the current prevalent ADV in China [19, 20]. In this study, numerous AA mutations were found in gB, gC and gD of Qihe547 ADV, especially in the dominant antigenic regions of gB (AA59-AA126 and AA507-AA734) [21].The heparan binding domains of gC (AA44-AA290) were also the antibody-binding domains [22]. As a result of AA variations, the potential antigenic peptides in gB, gC and gD were inconsistent between Qihe547 ADV and ADV Bartha. The differences of potential antigenic peptides in Qihe547 gB were mainly found in the dominant antigenic region (AA59-AA126, AA507-AA734). The extracellular region (AA23-AA453) in gC of ADV plays an important role in ADV adsorption, neutralizing antibody generation and virus neutralization. Many AA variations and the differences of potential antigenic peptides were observed in extracellular region in gC of ADV Qihe547. The differences of potential antigenic peptide were discovered in gD of ADV Qihe547. These antigenic peptide differences were found in the dominant antigenic region (AA59-AA126, AA507-AA734) of gB, the extracellular region (AA23-AA453) of gC, the dominant antigenic region of gD. The main protective antigen of Qihe547 ADV may be closely related with antigenicity of the virus, probably resulting neutralizing antibody differences between field strains and Bartha. And this may be the partial reason that the virus escapes from the vaccination of Bartha-K61 vaccine.

## Conclusion

In this study, high diversity was observed between Qihe547 and other ADV isolates (ADV32751/Italy2014, Kolchis, Bartha, Becker, and Kaplan). AA mutations were found in nearly each gene and unique AA indels were observed in 34 genes of ADV Qihe547. The AA variations and the differences of potential antigenic peptides in the important functional regions of the main protective antigen (gB, gC and gD) of ADV Qihe547 may contribute to immune evasion of the virus. The effect of the variations obviously requires further research.
